# Rare Presentation of Primary Pleural Ewing Sarcoma With a Mass Extending Into the Right Ventricle: A Case Report

**DOI:** 10.7759/cureus.57542

**Published:** 2024-04-03

**Authors:** Heba Alkoheji, Salman AlAli, Wael Tahseen, Mohamed Awadh, Ahmed T Shaarawy, Abdulrahman Almadani

**Affiliations:** 1 Internal Medicine, Bahrain Defense Force Hospital Royal Medical Services, Riffa, BHR; 2 Radiology, Bahrain Defense Force Hospital Royal Medical Services, Riffa, BHR; 3 Pathology and Laboratory Medicine, Bahrain Defense Force Hospital Royal Medical Services, Riffa, BHR

**Keywords:** extraosseous ewing's sarcoma, pleural ewing sarcoma, ewing sarcoma, extraskeletal ewing's sarcoma, adult

## Abstract

Primary pleural Ewing sarcoma is a rare type of Ewing sarcoma with only a few case reports identified in the literature. The condition is challenging to diagnose with deceiving symptoms and wide differential diagnosis. Diagnosis is confirmed with a combination of radiological and pathological assessment. Treatment is similar to other types of Ewing sarcoma with chemotherapy and surgery being the mainstay of treatment. We identify an unusual presentation of pleural Ewing sarcoma in a 31-year-old male with a mass extending into the right ventricular outlet causing rapid deterioration of the patient.

## Introduction

Ewing sarcoma is a rare type of aggressive tumor predominantly affecting adolescents and young adults. This malignancy is categorized into several types, including the conventional skeletal Ewing sarcoma, the extraskeletal variant, the malignant small cell tumor of the thoracic wall, and the primitive neuroectodermal tumors rooted in soft tissue. These tumors are unified by their origin in distinctive mesenchymal progenitor cells and are often characterized by the t(11;22)(q24;q12) chromosomal translocation, which is present in about 85% of cases. While extraskeletal manifestations in soft tissues are relatively rare, they present with unique clinical features and generally have a better prognosis compared to their osseous counterparts [1-3].

Even though Ewing sarcoma is the second most common primary bone malignancy in young adults, it only accounts for less than 5% of all sarcomas with extraskeletal Ewing sarcoma being a minority of these cases. Extraskeletal Ewing sarcoma commonly affects the head and neck, pelvis, paravertebral spaces, and lower extremities. However, other rare locations are also reported such as the skin, chest wall, retroperitoneum, orbit, and omentum [1,3,4]. 

The origin of Ewing sarcoma from the pleura is unusual. To our knowledge, specific prevalence data for primary Ewing sarcoma of the pleura are not found in the literature. However, the fact that Ewing sarcoma itself is rare and that extraskeletal Ewing sarcoma is a minority would indicate that it is exceedingly rare, especially since few case reports were found to the date of this report.

This case report details the medical journey of a 31-year-old male diagnosed with primary pleural Ewing sarcoma exhibiting an unusual extension into the right ventricular outlet, highlighting the complexities and aggressive nature of this rare disease manifestation.

## Case presentation

Clinical findings

A previously well 31-year-old male, presented to the emergency department of Bahrain Defense Force Hospital, reporting a three-week duration of productive cough with white sputum and intermittent blood streaks. He also experienced progressive wheezing and difficulty breathing during exertion and had lost 11 kg unintentionally over the past two weeks, accompanied by a reduced appetite. Additionally, he mentioned having night sweats but no fever or abdominal pain. It's important to note that he has a significant smoking history of 16 pack-years. Past medical history included gastroesophageal reflux disease (GERD) and irritable bowel syndrome (IBS), which was managed by chlordiazepoxide hydrochloride and clidinium bromide. He also had a surgical removal of a scalp epidermal cyst. He reported no known allergies. On examination, the patient was on room air and was not in respiratory distress. Auscultation of the chest revealed bilateral expiratory wheezes with equal bilateral air entry. Other systems examinations were unremarkable.

Diagnostic assessment

Laboratory

Upon investigations, a comprehensive set of laboratory tests were sent. All laboratory investigations were unremarkable, except for significantly elevated C-reactive protein and lactate dehydrogenase with levels of 107 mg/L and 597 IU/L, respectively, suggesting underlying inflammation. Iron studies revealed an inflammatory pattern with high ferritin. His complete blood count showed high white blood cells with levels of 11.6 x 10^9^/L and platelets of 531 x 10^9^/L. Tumor markers, prostate-specific antigen (PSA) and carcinoembryonic antigen (CEA), were sent and were negative. The D-dimer level was high with a value of 6 ug/ml. Microbiology tests were sent for urine legionella antigen, sputum culture, and sputum for acid-fast Bacillus (AFB), tuberculosis polymerase chain reaction (TB PCR), and respiratory panel, which were all negative.

Radiology

**Figure 1:**
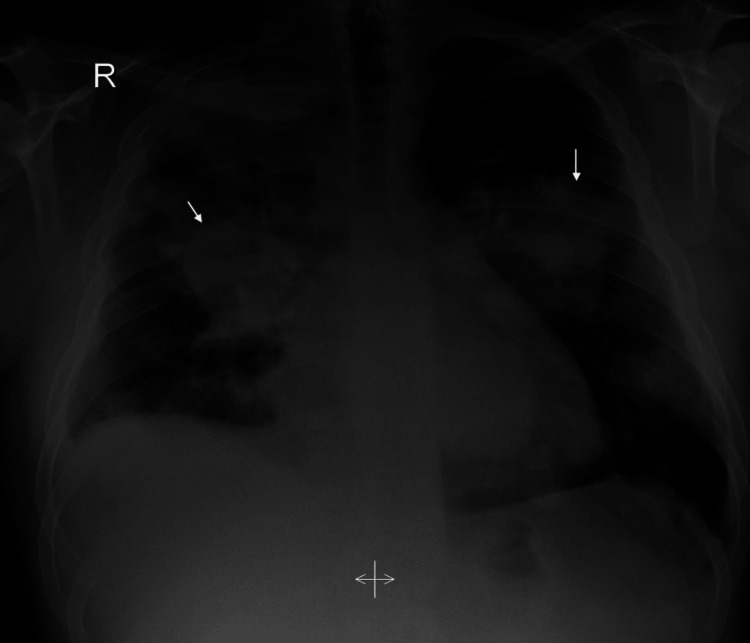
Chest X-ray: bilateral patchy dense opacities with marked hilar enlargement and a blunted right costophrenic angle

**Figure 2:**
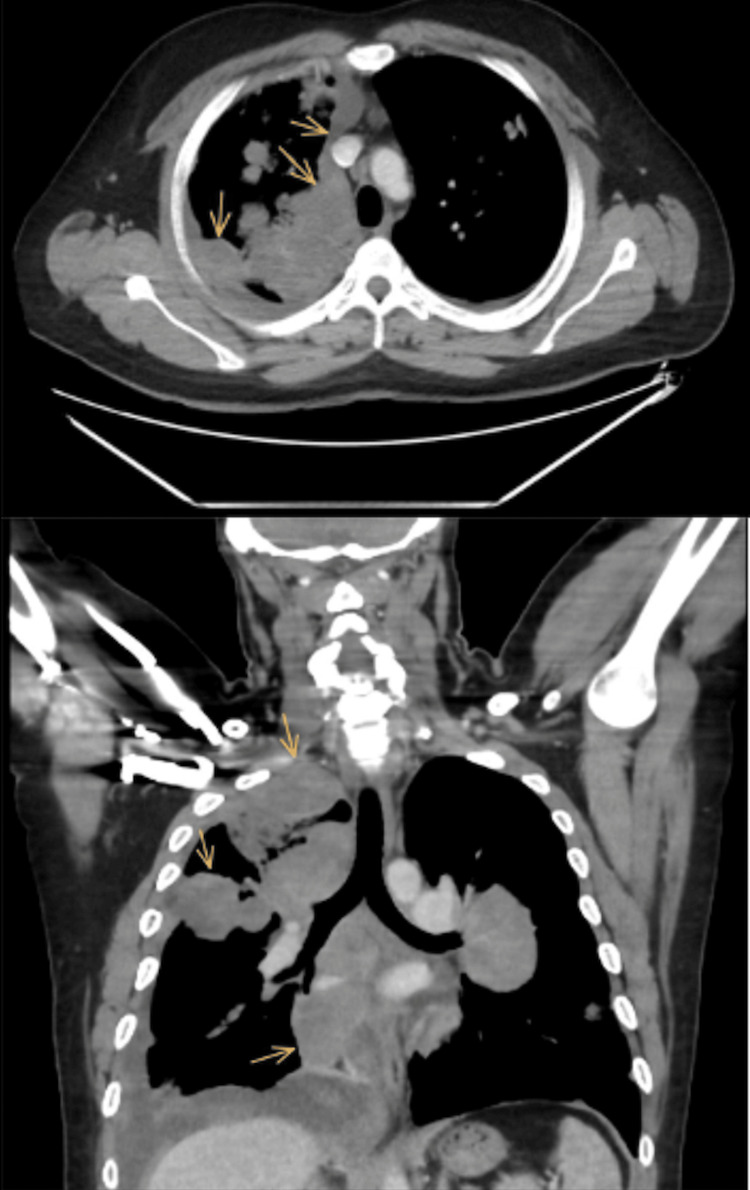
Axial and coronal CT chest with an IV contrast study showed pleural-based soft tissue pleural masses (yellow arrows) Computed tomography (CT); Intravenous (IV)

**Figure 3:**
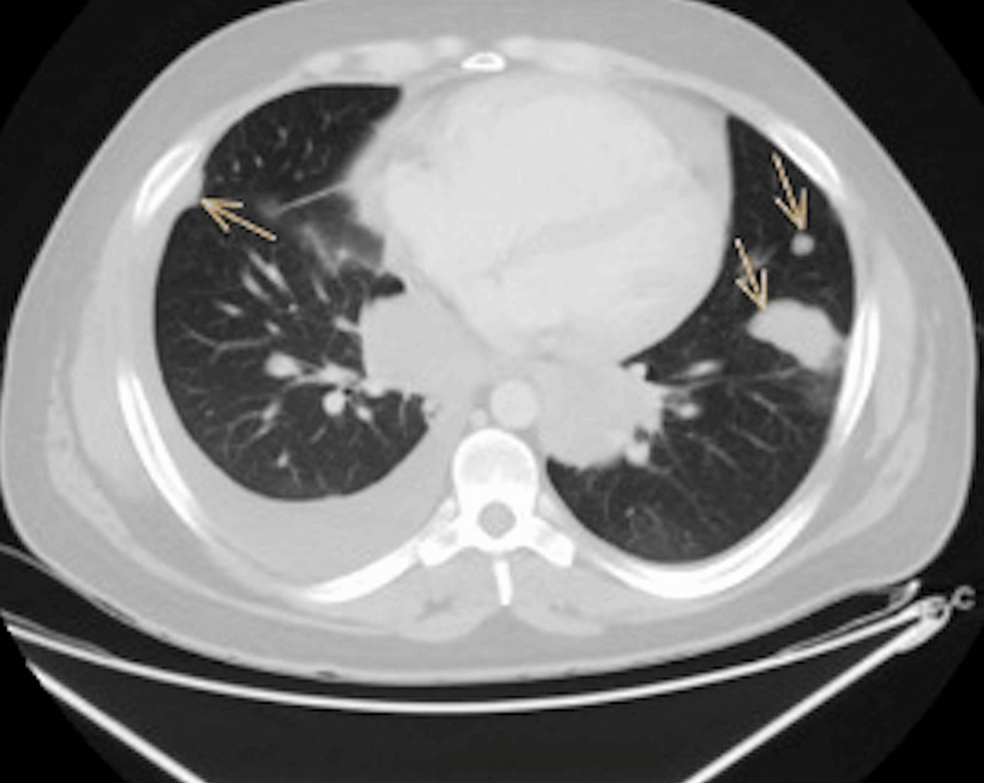
Axial CT chest with IV contrast study showed pulmonary parenchymal and pleural-based soft tissue metastasis. There is a pleural fluid collection on the right side. Computed tomography (CT); Intravenous (IV)

**Figure 4:**
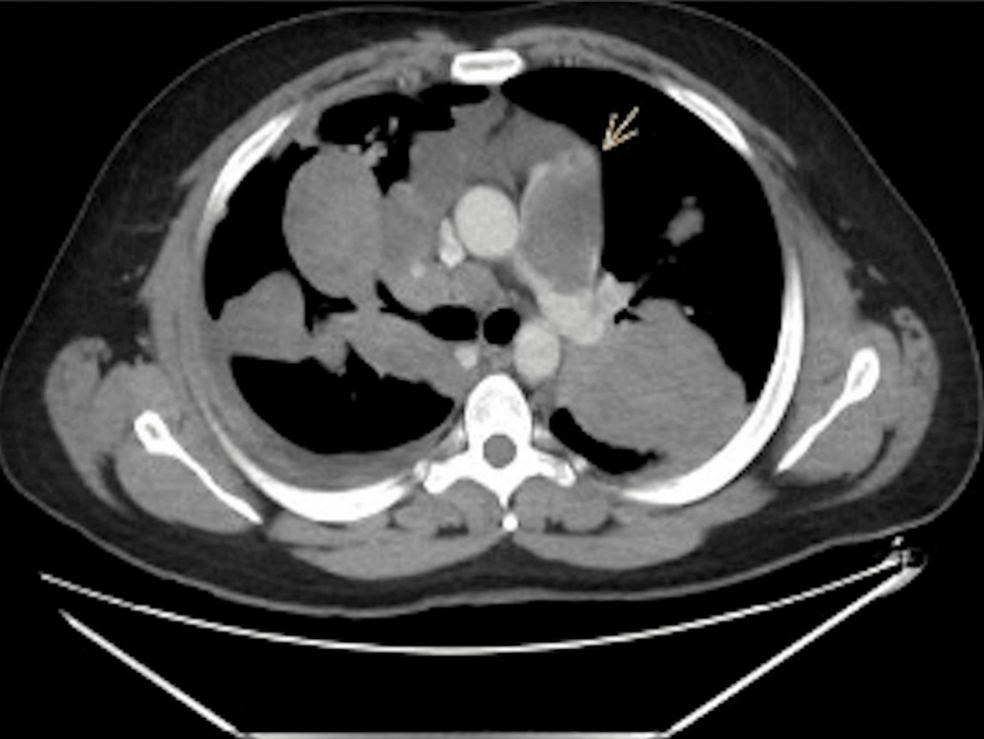
Axial CT chest with IV contrast study showed main pulmonary artery invasion by a soft tissue mass. CT-guided biopsy was taken from the left lung lesion. Computed tomography (CT); Intravenous (IV)

On admission, a chest X-ray was done, which revealed bilateral patchy dense opacities with marked hilar enlargement and a blunted right costophrenic angle (Figure 1). The X-ray was suspicious of cannonball versus bronchopneumonia. Thus, a computed tomography (CT) chest scan was performed using an intravenous contrast agent. It showed multifocal, multicentric pulmonary soft tissue masses predominantly adherent to the costal and mediastinal pleural reflections, more appreciated on the right side (Figures 2, 3). The lesions in the right upper lobe showed a wide base of attachments to the pleural reflections (Figure 2). Invasive main pulmonary artery tumoral thrombosis (Figure 3) and minimal right-sided pleural collection (Figures 3, 4). There is also multicentric nodular pleural thickening.

**Figure 5:**
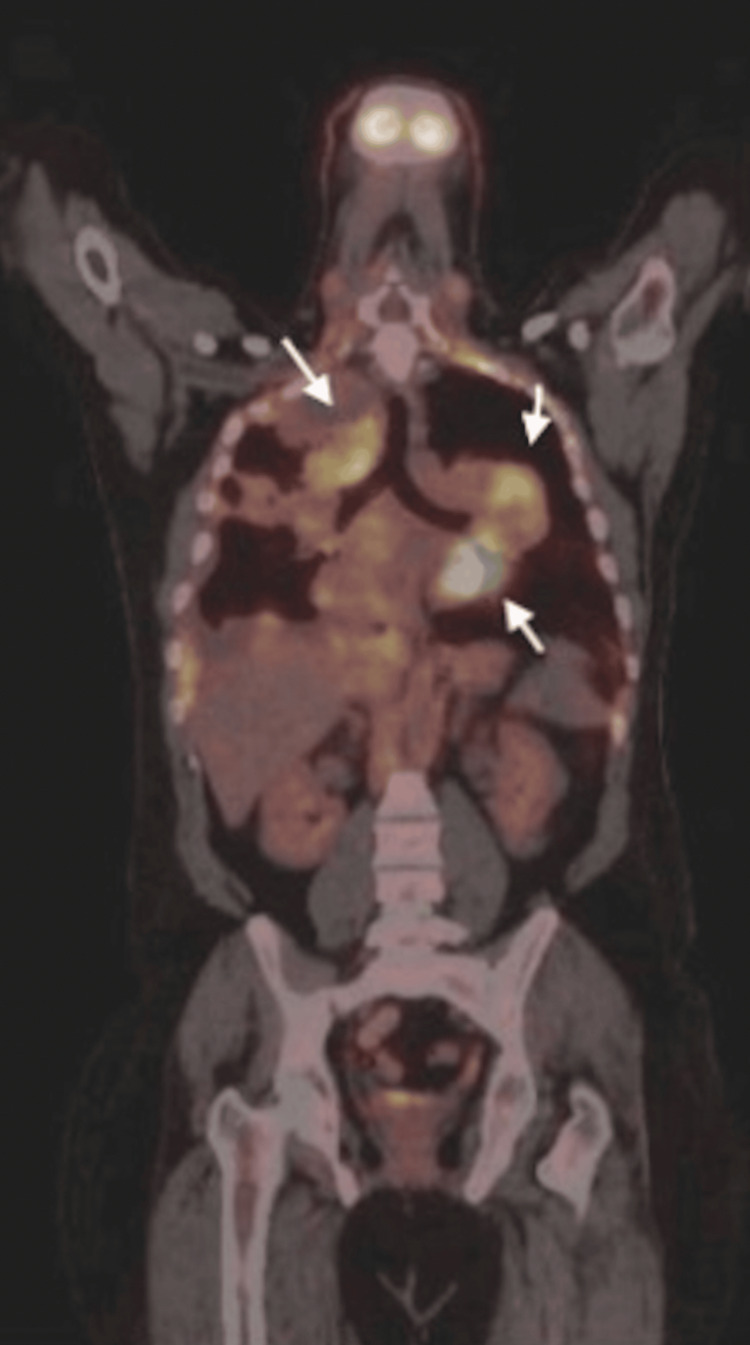
Coronal PET CT study showed multifocal FDG-18 vivid uptake in the pleural-based pulmonary soft tissue masses Positron emission tomography-computed tomography (PET CT); Fludeoxyglucose-18 (FDG-18)

CT-guided trucut tissue biopsy was taken from the left lower lobe pulmonary lesion (Figure 4). A positron emission tomography scan (PET) using the Fludeoxyglucose-18 (FDG-18) radioisotope showed avid uptake in the soft tissue lesions (Figure 5).

**Figure 6:**
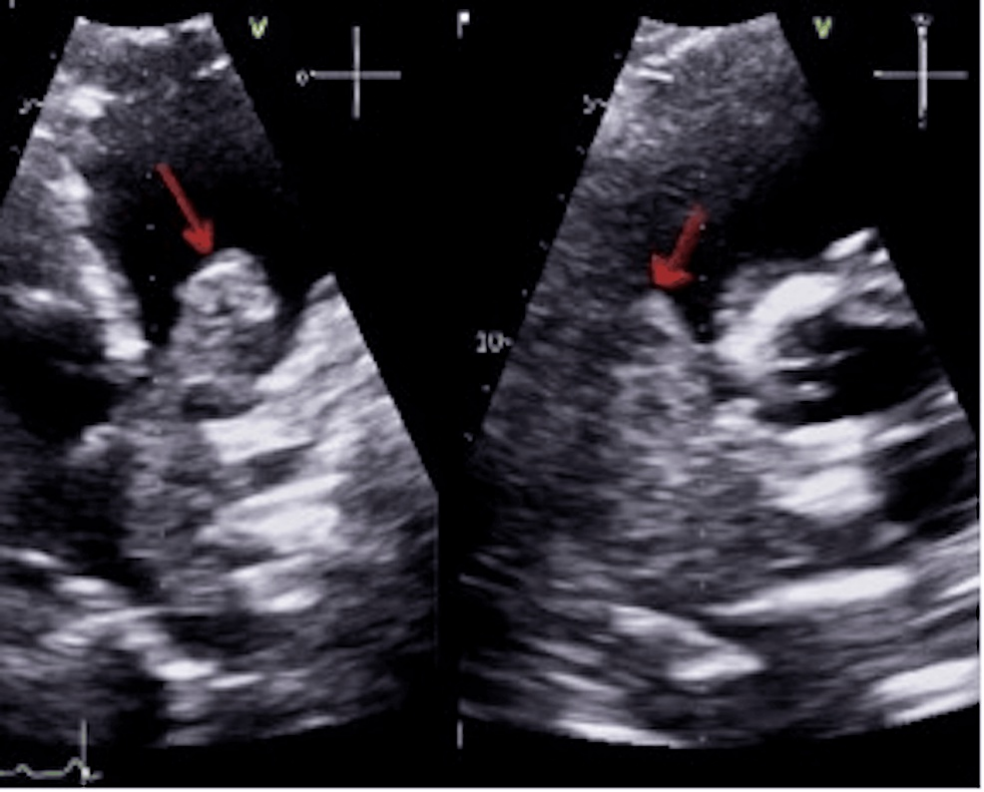
The echocardiography study showed a large hypoechoic mass inside the pulmonary artery extending to the right ventricle lumen (red arrows)

Moreover, US Doppler of the lower limbs was done in view of the pulmonary thrombosis, which showed deep vein thrombosis (DVT) over the left popliteal vein extending to the proximal anterior tibial vein and posterior tibial vein. Echocardiogram (ECHO) (Figure 6) findings showed a large hypoechoic mass inside the pulmonary artery extending into the right ventricular outlet causing almost complete obstruction of the lumen which includes the pulmonary valve, main pulmonary artery, and left pulmonary artery with the right ventricle strain. Repeated ECHO due to worsening of patient’s symptoms showed increased gradient across obstruction with severe right ventricular dysfunction.

Pathology

Pleural fluid analysis was done on presentation, which revealed exudative fluid but was negative for malignant cells. Following that, bronchoscopy and CT-guided co-axial lung biopsy showed a malignant tumor composed of relatively monomorphic spindle cells having lightly eosinophilic cytoplasm with an indistinct cell membrane arranged as sheets; short intersecting fascicles with frequent mitosis and foci of necrosis were also identified. The tumor infiltrated between fatty and skeletal muscle tissue. Immunohistochemistry showed the following in the tumor cells: positive for human melanoma black (HMB45), cluster of differentiation 99 (CD99), CD56, friend leukemia integration 1 transcription factor (FLI-1), and the homeodomain protein NK2 homeobox 2 (NKX2.2), and antigen Kiel 67 (Ki67) with the proliferation index being high, around 75%.

**Figure 7:**
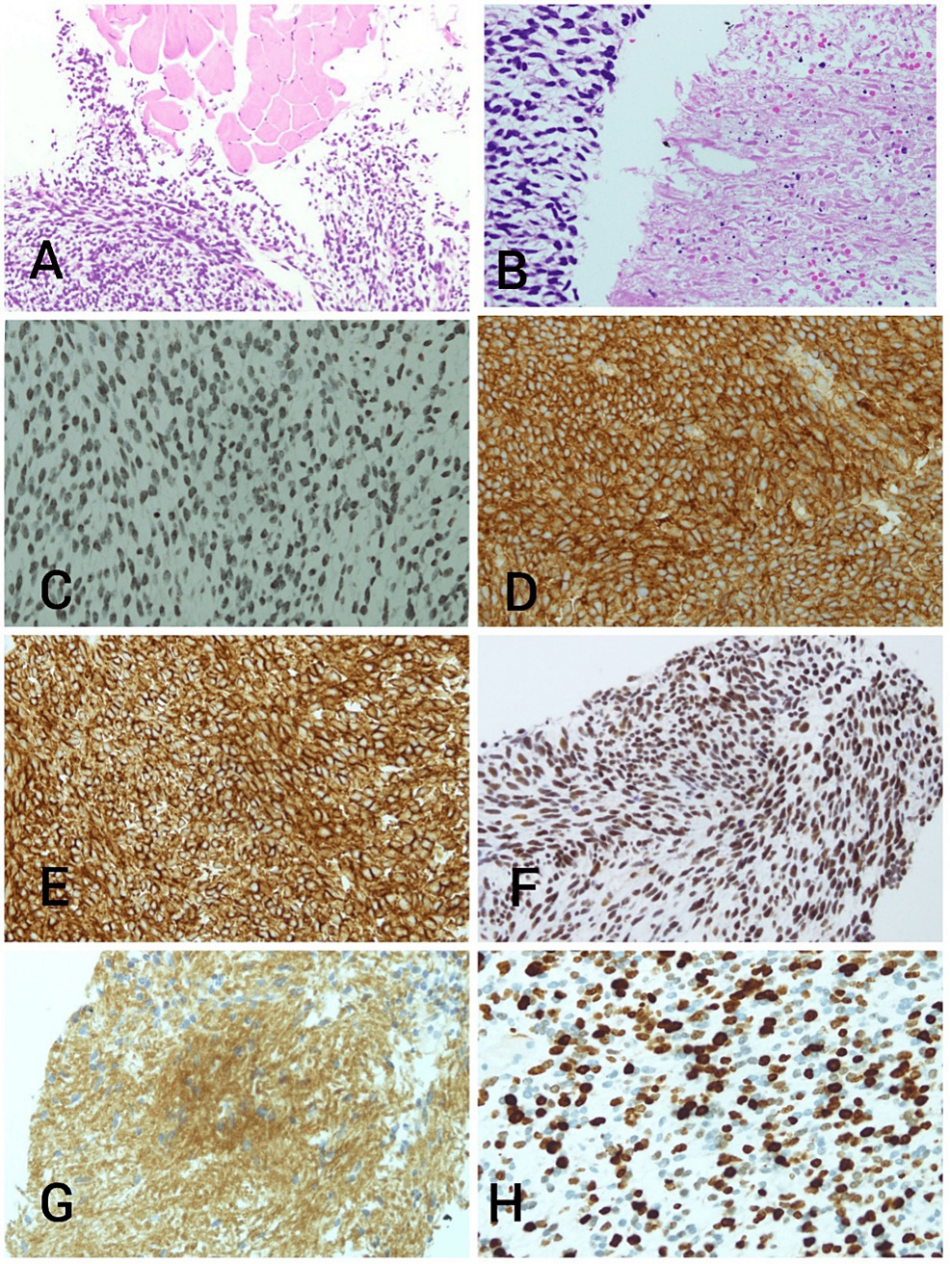
A: Low-power view showing a cellular spindle cell tumor infiltrate in between skeletal muscle bundles, B: High-power view of the tumor cells with an area of necrosis (right side of the image), C: HMB45, D: CD99, E: CD56, F: FLI-1, G: NKX2.2, H: Ki67 Human melanoma black (HMB45), cluster of differentiation 99 (CD99), cluster of differentiation 56 (CD56), friend leukemia integration 1 transcription factor (FLI-1), the homeodomain protein NK2 homeobox 2 (NKX2.2), antigen Kiel 67 (Ki67)

The overall appearances and immunohistochemical profile support the diagnosis of Ewings sarcoma/primitive neuroectodermal tumor (PNET) (Figure 7).

Hospital course

The patient's condition deteriorated quickly in less than one month of presentation. He was initially maintaining saturation on room air; however, the patient desaturated throughout admission requiring 10 Liters of oxygen prior to expiration. The patient was started on piperacillin-tazobactam 4.5 mg intravenous (IV) eight hourly and azithromycin 500 mg IV once a day, which was discontinued on day six of admission. The patient was kept on omeprazole 20 mg per oral (PO) once a day as gastric protection, to relieve his respiratory distress he was started on ipratropium bromide and albuterol 2.5 ml every four hours. Due to chest X-ray findings (Figure 1), CT with IV and oral contrast was done. Ultrasound aspiration of the pleural fluid was done due to the presence of pleural effusion, and analysis was sent as described earlier. Following the retrieval of the results, a bronchoscopy and CT-guided biopsy of the lung was done. Due to suspicion of pulmonary embolism in the CT scan, a US Doppler of the lower limbs was done.

The patient was initially kept on an enoxaparin sodium prophylactic dose but was switched to a therapeutic dose once deep vein thrombosis was confirmed. An echocardiogram was done, which showed surprising results as outlined above (Figure 6) and thus cardiothoracic surgery opinion was taken. Unfortunately, surgery has not been recommended due to the location and extent of the neoplasia and was deferred until tumor board discussion. A PET scan was performed (Figure 5), findings were as mentioned before, his case was discussed in the tumor board with an oncologist, cardiothoracic surgeon, vascular surgeon, and pulmonologist. Sadly, the patient was doomed to have a very poor prognosis and palliative therapy was recommended. The patient's condition was deteriorating and the patient coded and passed away with rapid deterioration in his symptoms.

## Discussion

Primary Ewing sarcoma of the pleura is a rare manifestation of Ewing sarcoma malignancy, with only a few case reports identified in the literature. Our patient was unique, the sarcoma had an extension to the right ventricle leading to his condition deteriorating very rapidly. Also, the diagnosis was challenging in the beginning, however, due to characteristic findings in the biopsy with immunohistochemistry showing CD 99 and CT findings supportive of neoplasia extending from pleura, primary Ewing pleural sarcoma was the final diagnosis.

In comparison to skeletal Ewing sarcoma, extraskeletal Ewing sarcoma usually has an older age of onset. In a literature review performed, the mean age of diagnosis of primary pleural Ewing sarcoma was found to be 16.6 years. The clinical presentation includes mainly chest pain, dyspnea, and cough, making it easily misdiagnosed as lung cancer. The size of the mass may also correlate with the symptoms. For instance, a large mass can invade adjacent pulmonary tissue causing symptoms like back pain, shortness of breath, and weakness. If the primary tumor is in the bone symptoms usually include bone pain that worsens at night. Of note, the presence of constitutional symptoms like weight loss and fever indicates advanced disease [1,5-7].

The diagnosis of primary pleural Ewing sarcoma involves a combination of clinical, radiological, and immunohistochemistry findings. A CT scan with contrast is usually sufficient for the diagnosis in such cases of Ewing sarcoma. A CT scan often shows a heterogenous enhancement with hypoattenuating areas corresponding to necrosis and a high-density focus in cases of hemorrhage may be evident in the affected areas. The appearance of pleural Ewing sarcoma tends to be enveloped and demarcated from nearby pulmonary tissue. Even though the tumor tends to metastasize via lymphatics. Lymphadenopathy is considered rare in extraskeletal Ewing sarcoma. It is challenging to accurately diagnose a patient with radiological imaging only, as it might overlap with other malignancies such as primary lung cancer. PET scans may be useful to monitor the disease activity and for surveillance purposes [6,7].

The definitive diagnosis can be confirmed via histopathological analysis with immunohistochemistry being crucial. The tumor is composed of small round blue cells closely adjacent packed cells in sheets with scanty eosinophilic cytoplasm. Focusing only on histological diagnosis with no immunohistochemistry might make the diagnosis challenging, as it has a wide range of differential diagnoses. Malignancies such as melanoma, lymphoma, rhabdomyosarcoma, squamous cell carcinoma, and small cell carcinoma can be difficult to differentiate from Ewing sarcoma. However, staining for CD 99 (p30/32) strongly indicates that the patient has Ewing sarcoma. Additionally, the presence of certain reciprocal translocations resulting in fusion genes such as t(11;22)(q24;q12) may help with the diagnosis of extraskeletal Ewing sarcoma [8].

The National Comprehensive Cancer Network recommended that all types of Ewing sarcoma be treated with the same approach. Systemic chemotherapy is a must to be started in all individuals with regimens including vincristine, dactinomycin, cyclophosphamide, and doxorubicin or vincristine, dactinomycin, and cyclophosphamide. The addition of ifosfamide and etoposide can be considered; however, no significant difference in survival rates was noted. The gold standard of treatment in extraskeletal Ewing sarcoma is surgical resection for localized disease. Chemotherapy can be neoadjuvant or adjuvant to surgery reducing relapses. The usage of radiotherapy is controversial. However, it is an effective option for unresectable localized tumors [3,9].

Given the rarity of the condition, we only found a few case reports within the literature that reported primary Ewing sarcoma of the pleura. The presentation varies among different cases but mainly presents with pleural effusion, as our patient and all confirmed the diagnosis with immunohistochemistry findings of CD99.

Xuexue et al. reported a case of a 14-year-old male presenting with fever and dyspnea. The origin of the extraskeletal Ewing sarcoma was reported to be in the parietal pleura causing massive pleural effusion. The metastasis was confined to the parietal and visceral pleura. Regarding the diagnostic approach, a CT chest with contrast was done followed by histological confirmation. The sample was taken through a thoracoscopic biopsy of the lesions. The patient also underwent fluorescence in situ hybridization, which showed rearrangement of 22q12. The patient received radiotherapy and chemotherapy with alternating regimens of vincristine, cyclophosphamide, adriamycin, etoposide, and ifosfamide [6].

Another 21-year-old patient presented with left massive pleural effusion with mediastinal shift. Interestingly, the female patient only presented with a fever; her vital signs were within the normal range. Unlike our patient, there was no metastasis to the lung. The diagnosis was made through a combination of a CT scan with contrast and histological confirmation. Biopsy was done through thoracoscopy [10].

An 11-year-old boy presented with different clinical symptoms as compared to the previous cases. His main symptom was left shoulder pain and exertional chest tightness. A combination of contrast-enhanced CT and MRI confirmed the presence of a pleural-origin tumor invading the left first rib. CT-guided biopsy was performed with histopathological analysis showing strong reactivity to CD99 and NKX2.2 Systemic chemotherapy was started with a combination of vincristine, cyclophosphamide, isocyclophosphamide, etoposide, and doxorubicin alternating regimens [7].

Until the date of this report, there are no cases showing extension to the right ventricle, which was unique to our case.

## Conclusions

Primary pleural Ewing sarcoma represents a rare entity in the general population with the condition having a poor prognosis. The identification of the disease may be challenging initially with vague presentation and wide differential diagnosis. Immunohistochemistry should be done to confirm the diagnosis. This case report identified a case of primary pleural Ewing sarcoma causing extension to the right ventricular outlet and leading to a poor outcome in a young adult.
